# The role of diagnostic, prognostic, and predictive biomarkers in the management of early pancreatic cancer

**DOI:** 10.1007/s00432-023-05149-4

**Published:** 2023-07-17

**Authors:** Sarah Maloney, Stephen J. Clarke, Sumit Sahni, Amanda Hudson, Emily Colvin, Anubhav Mittal, Jaswinder Samra, Nick Pavlakis

**Affiliations:** 1 Faculty of Medicine and Health Sciences, Northern Clinical School, The University of Sydney, Sydney, 2065 Australia; 2 Bill Walsh Translational Cancer Research Laboratory, Kolling Institute, The University of Sydney, Sydney, 2065 Australia; 3 Department of Medical Oncology, Royal North Shore Hospital, St. Leonards, Sydney, NSW 2065 Australia; 4 Upper Gastrointestinal Surgical Unit, Royal North Shore Hospital, St. Leonards, Sydney, NSW 2065 Australia

**Keywords:** Pancreatic, Cancer, Biomarkers, Prognostic, Predictive

## Abstract

Despite modern advances in cancer medicine, pancreatic cancer survival remains unchanged at just 12%. For the small proportion of patients diagnosed with ‘early’ (upfront or borderline resectable) disease, recurrences are common, and many recur soon after surgery. Whilst chemotherapy has been shown to increase survival in this cohort, the morbidity of surgery renders many candidates unsuitable for adjuvant treatment. Due to this, and the success of upfront chemotherapy in the advanced setting, use of neoadjuvant chemotherapy has been introduced in patients with upfront or borderline resectable disease. Randomized controlled trials have been conducted to compare upfront surgery to neoadjuvant chemotherapy in this patient cohort, opinions on the ideal upfront treatment approach are divided. This lack of consensus has highlighted the need for biomarkers to assist in clinical decision making. This review analyses the potential diagnostic, prognostic and predictive biomarkers that may assist in the diagnosis and management of early (upfront and borderline resectable) pancreatic cancer.

## Introduction

Pancreatic cancer continues to have poor overall survival, with 1 in 8 patients alive 5 years after diagnosis (Health and Welfare 2022). These outcomes have changed little in recent years despite more aggressive chemotherapy regimens and advanced surgical techniques. As the success of immunotherapy and tyrosine kinase inhibitors has seen the five-year survival rates of melanoma and lung cancer rapidly rise, pancreatic cancer survival remains on a plateau at just 12% (Health and Welfare 2022).

## Pathophysiology

**Fig. 1:**
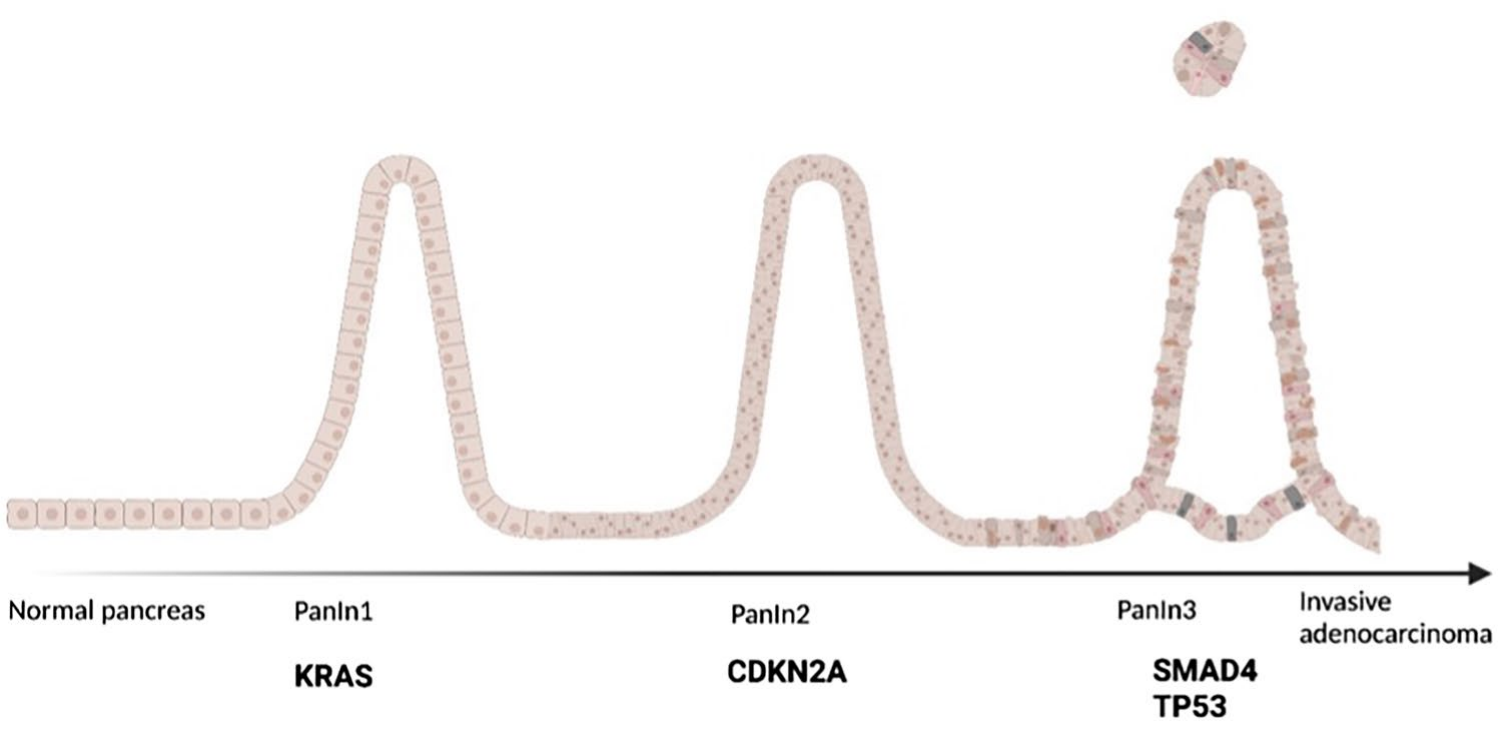
Oncogenes in the progression of pancreatic adenocarcinoma from pancreatic intraepithelial neoplasia (PanIN)

Pancreatic cancer can arise from precursor lesions which include pancreatic intraepithelial neoplasia (PanIN), intraductal papillary mucinous neoplasm (IPMN) and mucinous cystic neoplasm (MCN) (Hu et al. 2021). Of these PanIN occurs the most frequently and the pathogenic mutations which transform these lesions into an invasive adenocarcinoma are now well established. The most common oncogene mutations include KRAS, TP53, CDKN2A and SMAD4 (Fig. 1) (Buscail et al. 2020). KRAS mutations occur early in low grade (PanIN-1) lesions, whereas CDKN2A mutations occur as an intermediate event (PanIN-2), with SMAD4 and TP53 mutations occurring in late lesions (PanIN-3) (Luo et al. 2021).

## Diagnosis

A diagnosis of pancreatic cancer requires radiological evidence of a pancreatic lesion in combination with a histological confirmation, either through an endoscopic biopsy (EUS) of the lesion, or via common bile duct brushings from an endoscopic retrograde cholangiopancreatography (ERCP) (Yang et al. 2016). Due to the anatomical location of the pancreas and the risks associated with EUS and ERCP, sufficient tissue to allow for diagnosis can be difficult to obtain and multiple attempts may be required.

## Staging

Adequate staging is required in the diagnosis of pancreatic cancer to necessitate appropriate treatment. Minimum staging investigations involve computerised tomography of chest abdomen and pelvis (CT CAP) and may include magnetic resonance imaging (MRI) of the pancreas or positron emission tomography (PET) scanning. At diagnosis, pancreatic cancer presents as either localised or metastatic disease. Localised pancreatic cancer falls into three categories (upfront resectable, borderline resectable or locally advanced) and is determined by the tumour’s location to local vasculature as determined by pancreas focused CT. Tumours that have solid tumour contact with the superior mesenteric artery or the coeliac axis > 180° are termed locally advanced and are considered unresectable (Network 2019). The majority of patients have locally advanced or metastatic disease at the time of diagnosis (Health and Welfare 2022).

## Treatment

As surgery is not an initial option for patients with locally advanced disease, treatment consists of neoadjuvant chemotherapy, aiming to downsize the tumour and shrink it away from the adjacent blood vessels. For the few patients who have early (upfront or borderline resectable) disease at the time of diagnosis, treatment traditionally involved surgery followed by adjuvant chemotherapy; however, up to 30% of patients do not receive chemotherapy due to the morbidity associated with surgery (Mackay et al. 2020). This is of particular concern as patients who receive adjuvant chemotherapy have a significant improvement in 5-year survival, compared to those who do not receive chemotherapy (21 vs 8%) (Neoptolemos et al. 2004). Low rates of adjuvant chemotherapy administration in combination with the success of neoadjuvant chemotherapy in the locally advanced setting has led to an increased interest in neoadjuvant chemotherapy in patients with early (upfront or borderline resectable) disease. The most common chemotherapy regimens are either gemcitabine based (gemcitabine plus a fluoropyrimidine or gemcitabine plus nab-paclitaxel) or folfirinox (5-fluorouracil, irinotecan and oxaliplatin). The efficacy of gemcitabine plus abraxane versus folfirinox in the neoadjuvant setting in patients with resectable disease was compared in the SWOG S1505 trial, with similar two year survival in both groups (Sohal et al. 2021).

## Neoadjuvant chemotherapy versus upfront surgery in early pancreatic cancer

At present it is unknown whether the neoadjuvant therapy approach leads to superior outcomes compared with upfront surgery in early-stage patients. Three randomised controlled trials (RCTs) have been conducted to address this question. The PREOPANC trial, a phase III multicentre RCT which randomised patients with upfront or borderline resectable disease to receive either neoadjuvant treatment (gemcitabine based chemoradiotherapy) or upfront surgery. This study of 246 patients revealed an improvement in median overall survival in patients who received neoadjuvant chemoradiation (15.7 months) compared to the upfront surgical group (14.4 months) (Versteijne et al. 2022). In addition, patients in the neoadjuvant group were more likely to have a negative surgical margin, and there was no difference in surgical complication rates in the two groups (Versteijne et al. 2022). The PREP 02-JSAP 05 trial, a phase II/III RCT randomised 364 patients to neoadjuvant therapy consisting of gemcitabine + S1 to upfront surgery in patients with resectable disease. In this study, patients treated with neoadjuvant chemotherapy had a significant increase in median survival (36.7 months compared to only 26.6 months in the upfront surgical group) (Unno et al. 2019). In this cohort the resection rate, negative surgical margin rate and complication rates were comparable between the two groups (Unno et al. 2019). Finally, ESPAC-5F, a phase II RCT randomised 90 patients to upfront surgery or neoadjuvant treatment with either gemcitabine + capecitabine, folfirinox or chemoradiation in patients with borderline resectable pancreatic cancer. Overall survival at one year was superior in patients treated with either neoadjuvant gemcitabine plus capecitabine (78%) or folfirinox (84%) compared to upfront surgery (39%) (Ghaneh et al. 2023). There was no difference in surgical complication rates, surgical margin or rates of resection between patients treated with upfront resection versus neoadjuvant chemotherapy or chemoradiotherapy (Ghaneh et al. 2023).

Despite these promising results each of these studies had their own limitations. The overall survival difference in the PREOPANC study was clinically modest with only 1.3 months difference in the median survival between the two groups, although the hazard ratio did favour neoadjuvant chemoradiotherapy (0.73, 95% Confidence limit (CI) 0.56–0.96, p = 0.025). This study also utilised chemoradiation with gemcitabine, a modality yet to show survival benefit over chemotherapy alone in early pancreatic cancer. Regardless, the 5-year overall survival favouring the chemoradiotherapy arm was clinically meaningful (20.5% vs 6.5%), however the 5 year survival in the control arm was substantially lower than observed in the CONKO-001 trial of adjuvant gemcitabine (20.7%) (Oettle et al. 2013). The PREP 02/JSAP05 study used gemcitabine and S1 chemotherapy, a regimen not commonly used outside Japan. Finally, the ESPAC 5F trial was a small study of only 90 patients and allowed any chemotherapy to be used in the adjuvant setting, rather than a continuation of the chemotherapy used in neoadjuvant treatment.

In the absence of a large phase 3 randomised controlled trial supporting one treatment pathway over another the question remains open—should patients with upfront resectable and/or borderline resectable pancreatic cancer receive either neoadjuvant chemotherapy or upfront surgery?

A predictive biomarker to assist with this decision is long overdue.

## Diagnostic biomarker

The ideal diagnostic biomarker should be able to distinguish pancreatic cancer from benign pathologies such as chronic pancreatitis or precursor lesions such as IPMN or PanIN (Fig. 1).

## Prognostic biomarker

Treatment for early pancreatic cancer consists of both chemotherapy and surgical resection, which can have significant toxicity. The ability to differentiate which patients may have more aggressive disease, would assist clinicians to determine the optimal treatment pathway for each patient. At present, prognosis is determined by stage based on imaging (Network 2019).

## Predictive biomarkers

Due to the equipoise between neoadjuvant chemotherapy versus upfront surgery in early pancreatic cancer, biomarkers that may predict for response to chemotherapy may assist clinicians in determining which of the two clinical pathways is more appropriate for the patient in their clinic. In patients with an inherently chemoresistant tumour, early intervention with surgery may be more appropriate whereas in patients with a chemosensitive tumour early exposure to neoadjuvant chemotherapy may produce the best survival outcomes.

The challenge presented in the search for useful biomarkers in pancreatic cancer is that tissue obtained via biopsy at diagnosis is often scant, therefore an ideal biomarker must require small amounts of tissue, or be obtained via less invasive measures such as peripheral blood. In addition, biomarkers must be cost effective and accurate with an emphasis on a rapid turnaround time so that the results can be quickly factored into patients’ treatment. This is particularly important in early disease with the aim to start treatment soon after diagnosis.

In this review we summarise the literature on potential diagnostic, prognostic, and predictive biomarkers in the management of early (upfront or borderline resectable) pancreatic cancer (Table 1).

**Table 1:** Diagnostic, prognostic, predictive biomarkers in early pancreatic cancer

Diagnostic	Prognostic	Predictive
CA19.9	CA19.9	CA19.9
KRAS	KRAS	KRAS
TP53	TP53	
SMAD4	SMAD4	
CDK2NA	CDK2NA	CDK2NA
		BRCA
		MMR
	NLR, PLR, LMR	
	mGPS- Albumin, CRP	mGPS- Albumin, CRP
		hENT1
		GATA6
		MicroRNA21

## CA 19.9 (serum)

### Diagnostic, prognostic, predictive biomarker

Serum carbohydrate antigen (CA19.9), also known as sialyl-lewis A, is the most common tumour marker utilised in the diagnosis and management of pancreatic cancer (Table 1). It is commonly secreted in pancreatic malignancies as well as benign conditions of the biliary system including pancreatitis or pre-malignant lesions such as intraductal pancreatic mucinous neoplasm (IPMN) (Ballehaninna and Chamberlain 2012). It is related to the Lewis blood group and 10% of the population are Lewis antigen negative and do not secrete CA 19.9 (Ballehaninna and Chamberlain 2012).

Despite the high sensitivity and specificity of serum CA 19.9 in symptomatic patients ranging from 79–81% to 82–90% respectfully, the low prevalence of pancreatic cancer, makes it a poor screening and diagnostic test with a positive predictive value of less than 1% (Ballehaninna and Chamberlain 2012). Whilst its use as a single diagnostic biomarker is limited, the positive predictive value for diagnosis of pancreatic cancer is higher when combined with other clinical factors. Ritts et al. revealed that an elevated CA 19.9 combined with an image identified pancreatic mass, increased the positive predictive value to 94% (Ritts et al. 1994). Whereas, Tessler et al. demonstrated that the positive predictive value of CA 19.9 > 37 U/mL increased to close to 100% when associated with clinical and biochemical features including weight loss of more than 10 kg and hyperbilirubinemia (> 3 mg/dL) (Tessler et al. 2006).

CA 19.9 has also been assessed as a marker to assess stage, resectability status and hence prognosis in patients with a confirmed histological diagnosis of pancreatic cancer. Although the data is exclusively retrospective these studies do demonstrate promise, with higher levels of CA 19.9 associated with more advanced disease at diagnosis and hence worse prognosis (Ballehaninna and Chamberlain 2012). One such study by Humphris et al. analysed patients with early disease that underwent curative resection and discovered that patients with a pre operative CA19.9 < 120 U/ml had a significantly longer disease free survival compared to those with levels ≥ 120U/ml at 36 versus 17 months respectively (Humphris et al. 2012). Post-operative CA19.9 has also been investigated as a prognostic marker, with elevated CA19.9 > 37 U/mL associated with a poor prognosis and shorter overall survival then patients with a CA 19–9 ≤ 37 U/ml (12 versus 22 months) (Humphris et al. 2012).

Despite the abundance of retrospective reviews illustrating the potential of CA19.9 as a prognostic biomarker there are limitations to its use. At present optimal cut offs for CA19.9 vary between studies. Furthermore, the association between biliary obstruction and raised CA19.9 makes results difficult to interpret, as does the absence of this antigen in the 10% of patients with a Lewis antigen negative phenotype.

Preliminary studies analysing the role of CA19.9 have demonstrated its potential as a predictive biomarker in early disease. Humphris’ study of early pancreatic cancer patients, identified that post operative CA19.9 levels predicted for response to adjuvant chemotherapy (Humphris et al. 2012). In patients with a CA19.9 < 90 U/ml administration of adjuvant chemotherapy prolonged survival by nine months, whereas no survival benefit to adjuvant chemotherapy was observed in patients with CA19.9 > 90 U/ml (Humphris et al. 2012). Whilst extrapolation of CA19.9 as a biomarker to predict for neoadjuvant chemotherapy response based on this result is inappropriate, it is a hypothesis worth exploring in future prospective studies.

CA19.9 is the most versatile of biomarkers in treatment of pancreatic cancer, despite its lack of validation in all contexts. For patients with upfront or borderline disease, a disproportionately high CA 19.9 (in the presence of a normal bilirubin) may indicate the presence of micro-metastatic disease and hence treatment with systemic therapy early for these patients through the form of neoadjuvant chemotherapy may be more beneficial than upfront surgery.

## CEA (serum)

### Diagnostic, prognostic

Carcinogenic embryonic antigen (CEA) is a glycoprotein that is elevated in 30–60% of pancreatic cancer patients (Meng et al. 2017). As a diagnostic biomarker for pancreatic cancer, it has limitations with a sensitivity of only 43% and specificity of 82% in early or advanced disease (Meng et al. 2017; Zhang et al. 2015). Despite attempts at increasing sensitivity and specificity using various CEA based panels, the rates remain low, making it an unreliable diagnostic biomarker.

A meta-analysis discovered that for all pancreatic cancer patients (regardless if early or advanced) a higher baseline CEA was associated with a worse prognosis (Meng et al. 2017). Two retrospective reviews also demonstrated the use of CEA as a prognostic biomarker to discern between early or more advanced disease after standard staging. Both Van Manen and Fujioka et al. discovered that preoperative CEA was significantly higher in patients that had non curative and unresectable tumours at time of operation compared to those that had resectable, margin negative disease (Fujioka et al. 2007; van Manen et al. 2020). Although, validation in a prospective cohort is needed, the use of CEA as a prognostic biomarker in certain populations is promising.

There is minimal evidence that baseline CEA levels can predict for chemotherapy response in early pancreatic cancer patients. One retrospective review in patients with advanced disease demonstrated that patients that elevated CEA at baseline predicted for a worse response to palliative chemotherapy. Extrapolation of these results in patients with early disease is challenging as CEA is less likely to be elevated in that cohort (Boeck et al. 2013).

Like CA19.9, higher CEA levels at baseline may indicate more advanced disease, even in patients with radiological determined upfront or borderline resectable pancreatic cancer. As such, for patients with an elevated CEA at presentation, neoadjuvant chemotherapy, may be more appropriate than upfront surgery.

Oncogenes:

## KRAS (cytology, histology, plasma)

### Diagnostic, prognostic

*Kirsten Rat Sarcoma virus* (*KRAS*) gene encodes for the KRAS protein which is a crucial cell membrane protein involved in cell signalling, growth and differentiation (Karachaliou et al. 2013). When activated it promotes downstream signalling which leads to activation of the mitogen activated protein kinase (MAPK) pathway (Buscail et al. 2020; Cercek et al. 2020; Drosten and Barbacid 2020). A mutation of the KRAS protein results in this pathway being constitutively active, which leads to uncontrolled cell growth (Buscail et al. 2020). Up to 95% of pancreatic tumours contain a KRAS mutation, the majority of which involve a mutation in codon 12 (Bournet et al. 2016; Buscail et al. 2020; Waters and Der 2018). The most common KRAS mutation in pancreatic cancer is KRASG12D (Bannoura et al. 2022; Zorde Khvalevsky et al. 2013).

The role of KRAS in tissue in the diagnosis of pancreatic cancer has been debated. It often occurs early in carcinogenesis and is a common mutation in preneoplastic disease such as PanIN or IPMN (Buscail et al. 2020; Hu et al. 2021) (Fig. 1). It may be useful to discriminate between either invasive carcinoma or benign conditions including autoimmune pancreatitis (Hu et al. 2021). One prospective study demonstrated that the addition of KRAS testing on endoscopic ultrasound and fine needle aspiration (EUS-FNA) material increased the sensitivity, negative predictive value and accuracy of the cytopathology used to diagnosis pancreatic adenocarcinoma (Bournet et al. 2015). In this study KRAS mutations were identified in almost all samples (98%), thus making it a feasible addition to the cytopathology from the EUS (Bournet et al. 2015). At present, use of EUS FNA KRAS mutation testing is not part of routine clinical practice, in part due to lack of standardization of sampling and often insufficient tumour tissue obtained via endoscopic biopsy (Bournet et al. 2015).

In addition to its role as a potential diagnostic marker, many studies have examined the role of KRAS as a potential prognostic biomarker. Despite the discordant results, the majority of studies in patients with localised disease suggest the presence of a KRAS mutation in histological specimens correlates with a worse prognosis (Buscail et al. 2020).

KRAS as predictive marker (to targeted therapy) in pancreatic cancer is limited to the advanced or metastatic setting. Although present in only 1–2% of pancreatic adenocarcinoma, *KRASG12C* has recently been identified as a druggable target, with the introduction of a novel tyrosine kinase inhibitor, sotorasib (Strickler et al. 2022). In the CodeBreaK trial, patients of various malignancies with a *KRASG12C* mutation were treated with sotorasib. In the 38 advanced or metastatic pancreatic cancer patients enrolled, 21% had a partial response (Strickler et al. 2022). As such, KRAS may be useful as a predictive biomarker, specifically in the setting of KRAS targeting tyrosine kinase inhibitors, however this is limited to the advanced or metastatic setting. At present there is no role of KRAS in prediction of chemotherapy response and as such there is no role in identifying which patients may be more suitable for neoadjuvant chemotherapy or upfront surgery.

Due to the fact that insufficient tumour tissue for molecular testing is common in pancreatic cancer in addition to the high prevalence of KRAS mutations in pancreatic tumour tissues (> 90%), KRAS mutational status on circulating tumour DNA has also been assessed as a diagnostic and prognostic tool with varying levels of success (Brychta et al. 2016; Buscail et al. 2020; Zorde Khvalevsky et al. 2013). Early studies used polymerase chain reaction (PCR) methods performed on patient blood samples to assess for the presence of KRAS mutation in plasma of patients with confirmed pancreatic adenocarcinoma and in healthy controls (Brychta et al. 2016). In these studies, KRAS mutations in patients with confirmed adenocarcinoma plasma ranged from 27 to 35% (Brychta et al. 2016; Castells et al. 1999; Tada et al. 1993), compared with up to 72% of histological specimens. Although demonstrating early promise, the low sensitivity makes it an unreliable early diagnostic biomarker (Tada et al. 1993). Studies are conflicting regarding the role of KRAS in plasma as a prognostic biomarker (Brychta et al. 2016; Buscail et al. 2019; Castells et al. 1999). In part this conflict is due to variability of detection assays used to measure KRAS mutations in blood.

Validation studies are needed, however KRAS mutation testing may have a role in assisting in diagnosis in patients with early pancreatic cancer with sufficient tumour tissue on endoscopic ultrasound. In addition, the role of plasma KRAS has the potential as a non-invasive prognostic biomarker, once more sensitive assays are developed (Table 2).

**Table 2:** Genes, proteins, and RNA biomarkers

Genes	Proteins	RNA
	CA19.9	
KRAS	KRAS	
TP53	P53	
SMAD4	SMAD4	
CDKN2A	p16INK4A	
MLH1, MSH2, MSH6, PMS2	MLH1, MSH2, MSH6, PMS2	
	mGPS- Albumin, CRP	
	hENT1	
GATA6	GATA6	
		MicroRNA21

## TP53 gene (histology)

### Diagnostic, prognostic

*TP53* is the gene that codes for the p53 tumour suppressor protein and is mutated in approximately 50–75% of pancreatic cancers (Ansari et al. 2011; Morton et al. 2010; Talar-Wojnarowska and Malecka-Panas 2006). It inhibits cell proliferation and regulates programmed cell death (Biankin et al. 2002). Mutations in this gene can lead to uncontrolled cell growth and it is found later in pancreatic cancer pathogenesis (Fig. 1) (Kamisawa et al. 2016; Talar-Wojnarowska and Malecka-Panas 2006). It is infrequently mutated in benign conditions such as chronic pancreatitis however its low sensitivity for pancreatic adenocarcinoma makes it a poor diagnostic marker in early disease (Talar-Wojnarowska and Malecka-Panas 2006). Its use as a prognostic biomarker has also been assessed in small retrospective reviews. Of these only two identified a relationship between positive staining for p53 and a poor prognosis, however, only one study was in patients with early pancreatic cancer (Ahrendt et al. 2000; Linder et al. 1997). The majority of studies identified no role as a prognostic biomarker in patients with early pancreatic cancer (Ahrendt et al. 2000; Ansari et al. 2011; Linder et al. 1997; Morton et al. 2010). In vitro studies have demonstrated that the presence of a *TP53* mutation in cell culture resulted in chemotherapy (gemcitabine) resistance, however further in vivo studies are needed to validate these findings before it can be used as a biomarker to predict chemotherapy response (Nakamura et al. 2016; Ozaki et al. 2018).

## SMAD4 gene (cytology, histology)

### Diagnostic, prognostic

The *SMAD4* gene encodes for the SMAD4 protein, a transcription factor protein involved in the TGF beta pathway, which controls signal transduction from cell membrane to nucleus (Zhao et al. 2018). TGF beta has biphasic effects on tumour cell function. Initially it is involved in inhibition of tumour cell production by inducing cell apoptosis, however later in tumorigenesis it promotes epithelial to mesenchymal transition (EMT) which results in transition of the tumour cell into a more aggressive phenotype (Dardare et al. 2020; Hezel et al. 2006; Zhao et al. 2018). Due to its role as a tumour suppressor, mutations in SMAD4 result in reduction of cell apoptosis and increased cell proliferation.

SMAD4 is mutated in up to 55–60% of pancreatic adenocarcinomas and occurs later in tumorigenesis (Fig. 1) (Biankin et al. 2002; Zhao et al. 2018). The most common mutation being gene deletion (McCarthy and Chetty 2018). Mutations in SMAD4 are uncommon in benign conditions and immunohistochemical testing of SMAD4 on biopsy material, may prove to be a useful adjunct diagnostic marker, with loss of staining more likely to indicate invasive adenocarcinoma (Giannis et al. 2021; McCarthy and Chetty 2018).

The role of SMAD4 as a prognostic biomarker in patients with early disease is controversial. Two small studies demonstrate conflicting results with regards to SMAD4 mutations. A study conducted of 114 patients that underwent surgical resection demonstrated that patients with a mutated *SMAD4* gene had a worse median survival compared to those with wildtype *SMAD4* (Blackford et al. 2009*)*. Conversely, in a smaller study of 45 patients that underwent resection, a mutation in the *SMAD4* gene resulted in an improved overall survival (Biankin et al. 2002). To resolve this conflict, a meta-analysis involving patients with both early and advanced disease was conducted, in this analysis, patients with early disease, the presence of a *SMAD4* mutation was associated with worse prognosis and a hazard ratio of 1.5, however this was not evident in patients with more advanced disease (Shugang et al. 2016).

Preliminary cell culture studies, have revealed that *SMAD4* deficient cell lines have an increase sensitivity to cisplatin and irinotecan, thus offering a very early insight into SMAD4 mutations as a biomarker to predict for chemosensitivity (Cui et al. 2012). In addition, both in vitro and mouse studies have demonstrated that presence of SMAD4 mutation, may predict for radio resistance (Wang et al. 2018a, b). These studies have yet to be validated in humans.

## CDKN2A gene (histology)

### Diagnostic, prognostic, predictive

*Cyclin dependent kinase inhibitor 2A* (*CDKN2A*) gene encodes for two proteins p16^INK4A^ (p16) and p14^ARF^ with the former, the more common in pancreatic cancer (Chan et al. 2021). The p16 protein prohibits cell progression from the G1 to the S phase, hence arresting cell growth (Romagosa et al. 2011). Whilst *KRAS* mutations occur early in the pathogenesis of pancreatic cancer and *TP53* and *SMAD4* mutations occur later, *CDKN2A* mutations occur as an intermediate event (Fukushima et al. 2002; Romagosa et al. 2011) (Fig. 1). *CDKN2A* mutations occur in 30–50% of pancreatic cancer cases (Hayashi et al. 2017; Hu et al. 2021; Waddell et al. 2015).

A metanalysis was conducted to assess the role of *CDKN2A* mutations (in either tissue, blood, or pancreatic juices) in pancreatic cancer. In the 14 studies analysed, *CDKN2A* was significantly higher in patients with pancreatic cancer then in healthy controls (OR 17.2, p < 0.00001), however the low sensitivity (41%) and positive predictive value (58%) make it an unsuitable diagnostic biomarker (Tang et al. 2015).

Chen et al. assessed the potential of p16 as a prognostic biomarker in a retrospective analysis of 88 patients with early cancer treated with neoadjuvant chemoradiation (Chen et al. 2009). In this study the presence of a p16 mutation led to shorter recurrence free survival. Whilst promising, this was in contrast with several retrospective studies including a metanalysis by Gu et al., which found with no association between mutation and a worse prognosis (Chen et al. 2009; Du et al. 2022; Gu et al. 2020).

The evidence for *CDKN2A* as a potential predictive biomarker is limited to one retrospective review. Chen et al. analysed the role of *CDKN2A* as a predictive biomarker in patients with early (resectable) disease receiving gemcitabine chemoradiation and found that the presence of a *CDKN2A* mutation did not predict for treatment response (Chen et al. 2009; Du et al. 2022; Hong et al. 2021).

## DNA repair genes: BRCA 1 and 2 (histology, plasma)

### Predictive

DNA repair in the presence of cellular insult (exogenous or endogenous) is conducted by the homologous repair genes and up to 14.5–16.5% of patients diagnosed with pancreatic cancer, are found to have either a germline or somatic mutation in one of these genes (Casolino et al. 2021). The most frequent mutations are BRCA 1 and 2 (0.9 and 3.5%), ataxia telangiectasia (ATM 2.2%) and partner and localizer of BRCA2 (PALB2 0.2%) (Casolino et al. 2021). Germline mutations are tested using a plasma test, whereas somatic mutations are tested on tumour tissue.

The presence of a mutation in one of the homologous repair genes, predicts for response to drugs that induce double or single stranded breaks in DNA, the most common being platinum chemotherapy or poly-ADP ribose polymerase (PARP) inhibitors (Casolino et al. 2021). FOLFIRINOX, one of the two main neoadjuvant regimens used in pancreatic cancer contains oxaliplatin. The use of BRCA as a predictive biomarker in patients receiving platinum chemotherapy has been well studied in a range of different cancer types most notably breast and gynaecological malignancies (Gallagher et al. 2011). In the metastatic pancreatic cancer setting, the POLO1 trial investigated the role of a maintenance PARP inhibitor following treatment with a platinum chemotherapy in patients with a germline BRCA1 or BRCA 2 mutation (Golan et al. 2019). This study revealed a significant increase in progression free survival in patients treated with a PARP inhibitor vs placebo (7.4 vs 3.8 months), however, did not demonstrate increase in overall survival (Aguirre et al. 2018; Golan et al. 2019; Lowery et al. 2011; Wong et al. 2020).

Studies assessing the role of BRCA mutation to predict chemotherapy response in early (upfront and borderline resectable) pancreatic cancer are confined to small retrospective cohort analyses. In part this is due to a lack of testing in this population. One retrospective review of 61 patients with upfront or borderline resectable pancreatic cancer patients with known BRCA mutations, treated with neoadjuvant FOLFIRINOX, identified significantly higher rates of histological complete response in the patients with a germline BRCA mutation (44%) compared to the BRCA wildtype cohort (10%) (Golan et al. 2020). Patients with BRCA mutations also went on to have a longer disease free survival (Golan et al. 2020).

Although validation is needed, there is a potential role of BRCA as a predictive biomarker in patients with early or borderline resectable pancreatic cancer to help decide if upfront surgery versus neoadjuvant chemotherapy may be more appropriate. With an increased sensitivity to platinum chemotherapy, the presence of a BRCA mutation may make early treatment with a platinum chemotherapy (FOLFIRINOX) more appealing than upfront surgery. There are, however, limitations to its use. At present germline testing from plasma is offered only in patients with a significant family history and somatic testing on tumour tissue is limited to the advanced or metastatic setting.

## Mismatch repair proteins (histology)

### Predictive

The four genes and corresponding proteins that govern mismatch repair (MMR) in humans include MLH1, MSH2, MSH 6 and PMS2 (Marabelle et al. 2020). These proteins have been extensively studied in a range of cancer types, most prominent of which is colorectal cancer (Marabelle et al. 2020). Their function is to correct any DNA base mismatch that may have occurred during DNA replication (Li 2008). Loss of function of one or more of these proteins is termed MMR deficiency (dMMR) and occurs in 1–2% of patients with pancreatic cancer (Ghidini et al. 2020; Hu et al. 2018a, b; Luchini et al. 2021). Germline mutations of one of these proteins is seen in patients with Lynch syndrome. Unlike their MMR proficient (pMMR) counterparts, tumours that have dMMR develop hundreds of somatic mutations, which encode potential neoantigens (Marabelle et al. 2020). These neoantigens serve as a potential target for treatment with immunotherapy. Testing of MMR proteins in pancreatic cancer is performed on the tumour tissue either by immunohistochemistry or polymerase chain reaction (PCR) to detect amplified microsatellite loci (Chen et al. 2018; Zito Marino et al. 2022). Success of immunotherapy in targeting dMMR tumours became apparent through the results of the KEYNOTE 158 trial (Marabelle et al. 2020). This phase 2 single arm- pan tumour study assessed response of patients with dMMR tumours to pembrolizumab, after failure of standard therapy, highlighting the role of dMMR as a potential biomarker to predict response to immunotherapy. This study included 22 pre-treated stage IV pancreas cancer patients with dMMR and found an objective response rate of 18% with one patient undergoing a complete response (Marabelle et al. 2020). Although a single arm trial without comparator, these response rates are higher than expected in a heavily pre-treated metastatic pancreatic cancer cohort. At this stage, however, use of mismatch repair status to predict for immunotherapy response is restricted to advanced disease due to the lack of data in the early pancreatic cancer.

Preliminary studies have also been conducted assessing the role of mismatch repair status as a predictive biomarker for chemotherapy response with encouraging results (Sinicrope 2010). Riazy et al. demonstrated that in patients with early disease that underwent upfront resection the survival benefit of adjuvant therapy was dependent on the underlying mismatch repair status (Riazy et al. 2015). In patients with pMMR tumours, the addition of adjuvant chemotherapy significantly increased survival in patients that underwent surgical resection, however this survival advantage could not be appreciated in patients with dMMR tumours (Riazy et al. 2015). The lack of survival benefit in the dMMR cohort may reflect innate chemoresistance from dMMR tumours, a feature seen in colorectal cancer (Cercek et al. 2020). Although prospective studies are needed for validation, this early data may indicate the potential for dMMR as a useful predictive chemotherapy biomarker. For patients with early disease with dMMR staining, treatment with upfront resection may be more appropriate than treatment with neoadjuvant chemotherapy.

The literature surrounding use of mismatch repair as a prognostic biomarker is early disease is conflicting. Nakata Et al demonstrated a survival benefit in 46 pancreatic cancer patients that underwent resection and found that the 17% of patients with a mismatch repair deficient phenotype had a significantly longer survival time (Nakata et al. 2002). In contrast, Lupinacci et al. analysed 445 pancreatic adenocarcinoma patients and there was no survival difference in the 1.6% with dMMR (Lupinacci et al. 2018). In both studies, chemotherapy data was lacking.

## Inflammatory markers-NLR, PLR, LMR (plasma)

### Prognostic

Cancer associated inflammation is now an established hallmark of cancer (Colotta et al. 2009). Inflammation is a key risk factor in pancreatic cancer with a fivefold increase in risk of pancreatic cancer in patients with chronic pancreatitis (Kirkegård et al. 2017). Inflammation occurs initially through the innate immune system, as monocytes and neutrophils migrate to the damaged tissue and release proinflammatory cytokines (including tumour necrosis factor (TNF), interleukins and chemokines (Li et al. 2022; Waters and Der 2018). Many of these cytokines (CXCR1, 2 and 4) create a positive feedback loop and recruit additional neutrophils to the site of inflammation (Jin et al. 2021). After the initial immune response, the cells then activate the adaptive immune system which if it goes unchecked can lead to fibrosis and metaplasia (Li et al. 2022). After the tumour is established there is an ongoing dynamic relationship between the cancer and the body’s immune system, with inflammation a core component in the microenvironment of most neoplastic tissues (Colotta et al. 2009). In recent years the role of neutrophils in the promotion of carcinogenesis has become more apparent. In pancreatic cancer, high neutrophil infiltration into the tumour is associated more aggressive disease (Ino et al. 2013; Jiang et al. 2022). In one study of 102 patients, a high level of CD 177 (a protein associated with neutrophil activation) expression in pancreatic surgical specimens was associated with a significantly worse prognosis (Wang et al. 2018a, b).

In addition, a circulating neutrophil to lymphocyte ratio (NLR) has been studied to assess its role as a potential prognostic biomarker (Yang et al. 2015). A meta-analysis conducted on 1804 patients discovered higher NLR in patients with pancreatic cancer, was associated with reduction in overall survival, with a hazard ratio of 2.6 (Yang et al. 2015). This analysis was limited with only one study included involving patients with early disease, and this study demonstrated the predictive impact of high NLR on survival was attenuated with a hazard ratio of 1.2, and just meeting significance (p = 0.048). In comparison patients with advanced disease that underwent mixed modality treatment or patients that received palliative chemotherapy alone, higher NLR predicted for significantly shorter survival with a hazard ratio of 4.4 and 2.1 respectively indicating that NLR ratio may be of most value as a prognostic biomarker in patients with advanced disease (Yang et al. 2015). Stotz et al., similarly demonstrated this result and identified that a NLR > 5 in patients at baseline was associated with shorter survival both in patients with early disease that underwent surgery (HR1.6) and in patients with advanced disease (HR 2.5), with the hazard higher in patients with later stage disease (Stotz et al. 2013). Other inflammatory blood tests including platelet lymphocyte ratio (PLR) and lymphocyte monocyte ratio (LMR) have also been studied. Two meta-analyses have found that both higher PLR and lower LMR were associated with shorter survival, even in patients with upfront or borderline resectable pancreatic cancer (Hu et al. 2018a; Zhou et al. 2018).

There are limitations to the use of these inflammatory tests as potential prognostic biomarkers. Firstly, there is lack of consensus as to what a ‘high’ versus ‘low’ NLR cut-off should be with considerable heterogeneity between studies (Stotz et al. 2013; Yang et al. 2015). In addition, the levels of these inflammatory markers in the peripheral blood are often dynamic and change with other stimuli, including infection, chemotherapy, and supportive therapy (granulocyte-colony stimulating factor (G-CSF)).

## Modified glasgow prognostic scale (serum)

### Prognostic, predictive

Other key mediators in the cancer-inflammatory relationship are C reactive protein (CRP) and albumin. During an immune response, levels of CRP increase, and albumin levels decrease (Nurmi et al. 2021). Forrest et al. first established the Glasgow prognostic score (GPS) in 2003 (Forrest et al. 2003). Patients are accorded a score from 0 to 2, with 1 point for an elevated CRP (> 10 mg/l) and 1 point for a reduced albumin (< 35 g/l) (Forrest et al. 2003). This has since been updated to a modified GPS (mGPS). There have been several studies that have demonstrated that higher mGPS is associated with a poorer prognosis in patients with early disease. Yamada et al. analysed mGPS in resected pancreatic cancer patients and discovered that those a higher mGPS was associated with a median overall survival of only 17 months compared to 28 months in those with a low mGPS (Yamada et al. 2016). A meta-analysis revealed that higher GPS was strongly associated with worse outcomes in patients with pancreatic cancer, however survival difference was only evident in patients with advanced disease (Zhang et al. 2020).

At present, use of mGPS as a marker to predict chemotherapy response has not extensively been studied however one retrospective study of 56 patients revealed that in patients treated with neoadjuvant chemotherapy, a higher baseline mGPS was associated with a worse histological response (Hasegawa et al. 2016).

## hENT1 (histology)

### Predictive

Human equilibrative nucleoside transporter 1 (hENT1) is a transmembrane protein that transports nucleoside like drugs, into the cells (Giannis et al. 2021). In vitro studies have demonstrated that gemcitabine, utilises hENT1 to gain entry to cells where it has its cytotoxic effect (Muggia et al. 2012). In 2004 the first in vivo study was conducted to assess the relationship between the presence of hENT1 and response to chemotherapy (Spratlin et al. 2004). Shortly after, Spratlin et al. discovered that the absence of hENT1 was associated with poorer survival in patients with advanced pancreatic cancer (Spratlin et al. 2004). Studies conducted in early pancreatic cancer including subgroup analysis from RTOG9704 and ESPAC 3 trials confirmed these findings. In the RTOG9704 trial, in patients receiving adjuvant gemcitabine, the presence of hENT1 in the surgical specimen was associated with an improved overall survival and a hazard ratio of 0.5. For patients receiving 5 fluorouracil (an anti-metabolite chemotherapy), there was no difference in survival based on hENT1 expression (Farrell et al. 2009). Similar results were appreciated in the ESPAC 3 study with a hazard ratio of 0.6 for high hENT1 expression in patients treated with gemcitabine (Greenhalf et al. 2014). In contrast, one small study of 63 patients treated with neoadjuvant chemoradiation with gemcitabine, the presence of hENT1 expression in the surgical specimen was not associated with survival (Kawada et al. 2012). This lack of result may be due to testing on tumour that has already been exposed to gemcitabine, or due to the small sample size of only 63 patients.

In contrast Perera et al. analysed hENT1 expression on diagnostic biopsies (rather than surgical resections) in patients with advanced disease (Perera et al. 2022). Using RNA sequencing hENT1 high expression was associated with an improved overall survival in patients treated with gemcitabine, compared to hENT1 low expression, at 10.6 versus 6.7 months (p < 0.001). This survival advantage of hENT1 could not be appreciated in the patients that received FOLFIRINOX (Perera et al. 2022). As the treatment paradigm shifts towards more neoadjuvant therapies in pancreatic cancer, the role of hENT1 in biopsy samples as a predictor of chemotherapy response in patients with early disease will have to be further investigated.

## GATA6 (histology)

### Predictive

GATA 6 is a transcription factor, from the GATA6 gene (Martinelli et al. 2017). Although varying mechanisms have been proposed for the role of GATA6 in pancreatic cancer, the Collison et al. classification and subsequent molecular studies have demonstrated that GATA6 inhibits de-differentiation of epithelial to mesenchymal transition (EMT), a fundamental step in metastatic spread (Collisson et al. 2011; Martinelli et al. 2017). At present there are early studies demonstrating its role as a potential biomarker to predict response to 5 fluorouracil (5-FU), one of the key drugs utilised in management of early-stage disease (Martinelli et al. 2017). Martinelli et al. demonstrated in a cohort of ESPAC-3 patients that GATA6 expression strongly predicted for survival in patients treated with adjuvant 5-FU, with a median survival of 27 months in patients with high expression versus 14 months with low expression. This survival difference in GATA expression levels was not detected in patients treated with adjuvant gemcitabine (Martinelli et al. 2017). Other small retrospective cohort studies have demonstrated similar predictive capabilities of GATA6 (Andrés et al. 2021).

## MicroRNAs (histology)

### Predictive

MicroRNAs are small non coding RNAs that are involved in the regulation of gene expression (Hwang et al. 2010). In recent years, the emphasis has been on the role of miRNA in oncogenesis and chemoresistance (Frampton et al. 2015; Hwang et al. 2010; Vychytilova-Faltejskova et al. 2015). Of these studies, a number of miRNAs have been postulated to be involved in chemoresistance in pancreatic cancer, the most well established being miRNA21 (Hwang et al. 2010). Hwang conducted a retrospective analysis of early pancreatic cancer patients and found that high levels of miRNA21 expression was associated with reduced survival, in patients receiving adjuvant chemotherapy (either gemcitabine or 5FU) compared to patients with low expression (Hwang et al. 2010). This survival advantage was not evident in patients that did not receive adjuvant chemotherapy, suggesting its role in chemoresistance (Hwang et al. 2010). The potential for miRNA21 as a prognostic marker was also identified in a metanalysis, with high expression associated with increased hazard ratio of 2.6 (Frampton et al. 2015).

## Conclusion

In the absence of large, prospective phase 3 studies the optimal upfront treatment in patients with upfront and borderline resectable disease remains a contentious issue. Whilst neoadjuvant chemotherapy remains an attractive option in a disease is that is widely thought of as systemic from the onset, the ideal management strategy would be an individualised approach based on biomarkers. To be clinically effective, these biomarkers, would use minimal tissue to allow testing on diagnostic biopsy and provide rapid results and/or be blood based, to allow clinicians to choose between neoadjuvant chemotherapy or upfront surgery in a timely manner.

At present CA19.9 is the only validated biomarker in pancreatic cancer, used mainly for prognosis, however there are promising markers emerging that may be useful to assist in diagnosis, prognosis and to predict response to chemotherapy in early disease. The significant improvements in the cost and time to results in recent years of ‘OMICs’ testing, as well as improved techniques in tissue acquisition via endoscopic ultrasound, will further improve clinical utility of biomarkers in this terrible disease.
